# COVID-19 Research: Lessons from Non-Human Primate Models

**DOI:** 10.3390/vaccines9080886

**Published:** 2021-08-10

**Authors:** Laure Albrecht, Elodie Bishop, Basile Jay, Blaise Lafoux, Marie Minoves, Caroline Passaes

**Affiliations:** 1 Institut Pasteur, Centre d’Enseignement, Cours Virologie Fondamentale, 75015 Paris, France; laure.albrecht@ens-paris-saclay.fr (L.A.); elodie.bishop@ens.fr (E.B.); basile.jay@ens-paris-saclay.fr (B.J.); blaiselafoux@gmail.com (B.L.); marie.minoves@agroparistech.fr (M.M.); 2 Département de Sciences de la vie, Sorbonne Université, 75006 Paris, France; 3 École normale supérieure Paris-Saclay, 91190 Gif-sur-Yvette, France; 4 Département de Biologie, École Normale Supérieure, 75005 Paris, France; 5 Département de Sciences du vivant, Université de Paris, 75006 Paris, France

**Keywords:** COVID-19, SARS-CoV-2, non-human primates, pathogenesis, drug discovery, vaccine

## Abstract

Severe acute respiratory syndrome coronavirus 2 (SARS-CoV-2) is responsible for the coronavirus disease 2019 (COVID-19). It emerged from China in December 2019 and rapidly spread across the globe, causing a pandemic with unprecedented impacts on public health and economy. Therefore, there is an urgent need for the development of curative treatments and vaccines. In humans, COVID-19 pathogenesis shows a wide range of symptoms, from asymptomatic to severe pneumonia. Identifying animal models of SARS-CoV-2 infection that reflect the clinical symptoms of COVID-19 is of critical importance. Nonhuman primates (NHPss) correspond to relevant models to assess vaccine and antiviral effectiveness. This review discusses the use of NHPs as models for COVID-19 research, with focus on the pathogenesis of SARS-CoV-2 infection, drug discovery and pre-clinical evaluation of vaccine candidates.

## 1. Introduction

Coronaviruses (CoVs) correspond to a large group of positive-stranded RNA viruses that were first identified in the 1960s. Since then, seven coronaviruses have been identified to cause infections in humans. The coronaviruses 229E, OC43, HKU1 and NL63 are common in the human population and are typically responsible for seasonal respiratory infections [1]. Since the beginning of the 21st century, however, three highly pathogenic coronaviruses with zoonotic origin resulted in human outbreaks. In 2002 and in 2012, the severe acute respiratory syndrome coronavirus (SARS-CoV) and the Middle-East respiratory syndrome coronavirus (MERS-CoV) caused more than 8000 and 2500 cases, with 774 and 866 deaths, respectively [1]. In December 2019, the severe acute respiratory syndrome coronavirus 2 (SARS-CoV-2) was identified in a cluster of patients afflicted with a respiratory disease from viral etiology in Wuhan, China, which then became a pandemic [2]. In April 2021, more than 138 million cases and 2.9 million deaths were reported worldwide [3]. In humans, SARS-CoV-2 infection produces symptoms ranging from mild flu to a severe acute respiratory infection, a syndrome termed coronavirus disease 2019 (COVID-19) by the World Health Organization (WHO). SARS-CoV-2 is transmitted through aerosols, droplets and contact with infected people or contaminated surfaces [4]. Stringent strategies, such as lockdowns and curfews, had to be adopted to mitigate SARS-CoV-2 spread, which have an unprecedented impact on the global economy and long-term psychosocial consequences [5,6]. The emergence of SARS-CoV-2 variants with potential to spread faster and to impact disease severity [7] urges for the rapid development of innovative treatments and accessible vaccines to contain this pandemic.

The identification of suitable animal models is necessary to explore the mechanisms of pathogenesis and to develop countermeasures against SARS-CoV-2. Among these, non-human primates (NHPs) represent a highly valuable alternative for the study of the mechanisms underlying human viral infections. NHPs are phylogenetically related to humans and share a wide range of viral pathogens, often mimicking the clinical presentation of human infections [8]. In addition, their immune system, respiratory system anatomy, and tissue structure are very similar to those of humans. This review focuses on the relevance of NHPs as models for COVID-19 research.

## 2. NHP Models for SARS-CoV-2 Infection

Several NHPs species were previously investigated in the context of SARS-CoV and MERS-CoV infections, particularly Rhesus macaques (RhM-*Macaca mulatta*), Cynomolgus macaques (CyM-*Macaca fascicularis*), African Green monkeys (AGM-*Chlorocebus sabaeus*) and Common marmoset (*Callithrix jacchus*) [9,10,11,12,13,14,15,16]. Based on previous reports, these NHPs were investigated as possible models for SARS-CoV-2 pathogenesis [17,18,19,20,21,22]. Likewise, baboons (*Papio* sp.) were also studied for their susceptibility to SARS-CoV-2 infection [23].

Overall, different NHP species exhibit heterogeneous spectrum of SARS-CoV-2 infection [18,23]. To date, RhM and CyM are the species best characterized for COVID-19 drug and vaccine research [17,24]. In general, SARS-CoV-2 infection in macaques recapitulates the histological abnormalities and clinical manifestations observed in humans [2]. Of note, RhM presents stronger immune responses and more severe clinical signs when compared with CyM [18]. However, one major caveat is that SARS-CoV-2 infection in both RhM and CyM only resembles mild to moderate cases in humans.

Common marmosets showed a lower susceptibility to SARS-CoV-2 when compared to other NHP species [18,23]. Some studies suggested that aged-AGMs and baboons present a more severe respiratory disease and longer viral shedding than RhM, making them good candidates to model severe human infections and to test antiviral therapies [19,23,25,26,27]. In addition, baboons are the preferred NHP model for cardiovascular and metabolic diseases, which may allow the study of COVID-19 associated with comorbidities [23]. The association between age and disease severity described in humans is observed in all susceptible NHP species [23,25,26,28]. The features of SARS-CoV-2 pathogenesis in NHPs are summarized in Table 1 and are discussed in detail in the following sections.

### 2.1. SARS-CoV-2 Replication, Shedding and Distribution in Respiratory Tract and Other Tissues

Following transmission through aerosols, droplets and contact with infected people or contaminated surfaces, SARS-CoV-2 enters target cells by the interaction between the spike glycoprotein present in the surface of viral envelope and its cellular receptor. Angiotensin I converting enzyme 2 (ACE-2) was identified as the main cellular receptor for SARS-CoV-2 [30]. ACE-2 is mostly expressed in airway epithelial cells, lung parenchyma and vascular endothelial cells in the kidney and small intestine [31]. ACE-2 expression is a critical factor determining host susceptibility to the virus. All NHP species studied were susceptible to SARS-CoV-2 infection, as demonstrated either by live virus titration, RT-qPCR or, indirectly, by Ig titers [18,19,23]. As mentioned previously, common marmosets were the less susceptible. The lower susceptibility of marmosets may be partially explained by four amino acid changes in the ACE-2 receptor at positions critical for the interaction with SARS-CoV-2 spike, whereas RhM, CyM and humans share the same sequence [18]. The single cell transcriptomic atlas of CyM tissues explored the expression profile of ACE-2 and transmembrane serine protease 2 (TMPRSS2), the two major factors enabling viral entry. This study evidenced that ACE-2 expression in tissues differs between human and CyM, especially in lung and kidney, which may impact disease pathogenesis. TMPRSS2 distribution was similar in cell subtypes of lung, kidney and liver between human and monkey [32].

Collectively, viral kinetics in the lungs is similar in the most susceptible NHP species upon SARS-CoV-2 inoculation. They typically developed high viral loads in both upper and lower respiratory tracts, with a peak around 2–3 days post-infection (dpi), followed by a decrease to undetectable levels by 14 dpi [18,21,22,23,25,26,28]. All NHP species recapitulate the influence of age observed in humans because aged monkeys had higher viral titer peak and lower clearance rates [18,19,21,23,28].

While the highest viral titers were found in the respiratory tract, dissemination to extra-respiratory organs such as lymph nodes, kidneys, liver, spleen, heart, digestive and urinary tracts, and testicles is often observed [22]. A high number of viral genome copies can be found in secondary lymphoid organs early after inoculation, indicating that viral replication may occur in lymphoid tissues [22]. Several studies evidenced an impact of SARS-CoV-2 infection in the gastrointestinal tissues, suggesting a role in pathogenesis and transmission. Live viral shedding through respiratory and gastrointestinal tracts was found to last as long as 28 days in some cases [22]. Interestingly, intragastric inoculation with SARS-CoV-2 resulted in the productive infection of digestive tissues and inflammation in both lung and digestive tissues in RhM [33]. Some studies have indicated that viral RNA concentrations in wastewater or sewage may correlate with and even predict COVID-19 cases [34,35,36,37]. Hence, the fecal–oral route may be involved in SARS-CoV-2 transmission and must be taken into account for disease containment strategies.

Altogether, these studies evidenced that SARS-CoV-2 can disseminate and replicate in multiple tissues in susceptible NHP species and remain infectious for several weeks. This might raise questions about the potential of this virus to persist in their organism. In humans, there are increasing reports of persistent and prolonged symptoms after acute COVID-19 [38,39]. Whether SARS-CoV-2 has the capacity to persist in different tissues and the long-term impact of this persistence are yet to be investigated. For this purpose, studies in NHPs are of major interest.

### 2.2. Clinical Manifestations and Histopathological Abnormalities upon SARS-CoV-2 Infection

The NHP models recapitulate several clinical features of mild to moderate COVID-19 cases in humans. The most consistent observations were asthenia, body weight loss and loss of appetite [18,19,40]. Dyspnea, abnormal respiratory patterns and mild hypoxia were occasionally described, particularly in RhM [25,29,40]. Lethality associated with SARS-CoV-2 infection was not reported in NHP models (except in one study, in which lethality was reported in two aged AGMs [28]).

The histopathological abnormalities observed in the lung of COVID-19 patients are also observed in NHPs. Chest radiographs and lung histopathology evidenced common features observed following SARS-CoV-2 infection: pulmonary infiltrate, diffuse alveolar damage and hyaline membrane formation [2]. Radiological alterations (including ground-glass opacities, infiltrates and obscure pulmonary vascular markings) were consistently observed and showed good correlation with disease severity [24,26,29,41]. Notably, CT scans and PET/CT combination showed valuable information in evaluating lesions severity and their evolution throughout the course of the disease in COVID-19 patients and in all NHP models [42]. 

At necropsy, clear signs of pathological changes were observed in the organs of SARS-CoV-2-susceptible NHPs. On the macroscopic scale, lung tissue could present focally discolored and consolidated, typical of organ failure and collapse [19,28,29]. Macroscopic lesions of the lungs were accompanied by overt microscopic changes characterizing pneumonia, frequently concentrated on terminal bronchioles [9,28,29]. Alveolar septa were thickened, with an increased number of monocytes in the alveolar cavities [29]. The interstitium was infiltrated with a high proportion of immune cells, such as macrophages and T lymphocytes [43], which may cause occasional perivascular lymphocytic cuffing. Necrosis was observed in severe lesions, characterized by the degeneration of epithelial cells and macrophages, leading to hyaline membrane formation, being associated with alveolar flooding, which compromises gas exchanges [21,29,40]. Regeneration of the damaged epithelium was characterized by the presence of hyperplasia of type II pneumocytes [21,40]. Viral cytopathic effects such as multinucleated syncytial cells were rare [28,29]. 

Immunohistochemistry assays revealed the presence of viral antigens in type I and type II pneumocytes and in monocytes of the alveolar cavity of susceptible NHP species [21]. Viral antigens were also detected in extra-respiratory organs, notably in lymphoid organs such as bronchus-associated lymphoid tissue (BALT), bronchial and mediastinal lymph nodes [22,44]. In addition, viral antigens were also found in the lamina propria of the gastrointestinal tract, which is in line with viral detection and shedding through this system.

So far, data of blood biochemistry analysis are scarce in SARS-CoV-2 NHP studies. Blood urea nitrogen (BUN) and amylase levels were decreased, while the levels of hepatic enzymes were elevated in AGM and CyM [29]. Decreased serum albumin and hemoglobin levels, and progressive increasing total serum CO_2_ levels, which are indicators of pulmonary dysfunction, were observed in RhM.

Anemia and thrombocytopenia were observed early following viral infection, probably as a consequence of lung damage and inflammatory response [19,23,29]. 

With the exception of one study that reported acute respiratory distress syndrome (ARDS) in two aged AGMs [28], overall, clinical signs of SARS-CoV-2 infection in NHPs correspond to mild/moderate forms of COVID-19. However, the clinical manifestations of SARS-CoV-2 infection in these NHP models allow us to elucidate disease pathogenesis and to evaluate treatments and vaccines. 

### 2.3. Cellular Alterations following SARS-CoV-2 Infection

Due to their similarity with the human immune system, NHP models are of particular interest to explore the cellular alterations in blood and tissues following SARS-CoV-2 infection [8]. After infection with SARS-CoV-2, the body responds by initiating a rapid immune response involving the activation of different immune cells. 

In the blood of NHPs, the early phase of SARS-CoV-2 infection was characterized by a transient increase in monocytes, myeloid dendritic cells (mDC), with a peak around 2–4 dpi, then followed by leucopenia. Natural killer cell (NK) levels seem to decrease over the course of the infection [22,43,45,46]. Some studies reported early variations in blood neutrophils following SARS-CoV-2 infection [23,25,45], but others did not observe significant changes [23,43]. 

In the lungs, the early response against SARS-CoV-2 infection is characterized by the recruitment of neutrophils, monocytes, NK, and plasmacytoid dendritic cells (pDC) [25,27,43,45]. This intense recruitment of immune cells to the lungs was observed in all NHP species. As part of the initial response against SARS-CoV-2, these cells secrete pro-inflammatory cytokines, contributing to local inflammation, a hallmark of COVID-19 pathogenesis [2]. In infected RhM and AGM, the accumulation of monocytes and neutrophils in the lungs was associated with severe disease. Animals with anti-inflammatory responses had less severe manifestations [43].

With regard to the dynamics of T and B lymphocytes, some studies reported an increase in T cells around 3 dpi, which was then followed by lymphopenia, likely due to the migration of CD4+ and CD8+ T cells to the sites of viral replication. B cell numbers also decreased rapidly after infection and then rebounded over the next several weeks in blood. Following the increase in viral load in the respiratory tract around 5–7 dpi, the number of T and B lymphocytes concomitantly augmented, suggesting a key role for cellular and humoral responses in the control and resolution of SARS-CoV-2 infection [22,23,43,44,45,46,47,48]. Overall, differences in the cellular dynamics associated with age were not reported in NHPs [23].

Altogether, the dynamics of innate and adaptive immune cells reflect the establishment of a rapid and coordinated acute response against SARS-CoV-2 infection. An impaired or delayed dynamics of these immune cells may have potential implications in disease severity.

### 2.4. Cytokine Storm Contributes to COVID-19 Pathogenesis

A rapid and effective innate immune response is crucial as a first-line defense against SARS-CoV-2. Ineffective innate responses may result in abnormally high levels of cell activation and pro-inflammatory cytokines and chemokines [49]. In the first days following SARS-CoV-2 infection, several cytokines were consistently found to be elevated in the plasma of infected NHPs. Increased levels of IFNα, IFN-γ, IP-10, IL-1Ra, IL-2, IL-4, IL-5, IL-6, IL-15, TNF-α, MCP-1 and Eotaxin were reported [2,25,27,43,50,51].

Studies in NHPs allowed a longitudinal characterization of local cytokines and chemokines secreted in the lung tissue [22,23,25,43,50]. In a resolved SARS-CoV-2 infection, three waves of cytokine production were observed. Within 3 dpi, there is an increase in the levels of IFNα, IFN-γ, IP-10, IL-1β, IL-1Ra, IL-5, IL-6, IL-8, IL-12, IL-18, MIP-1α, MIP-1β, Perforin and TNF-α, reflecting the activation of local innate immune responses, the recruitment of immune cells such as neutrophils, monocytes, NK and pDCs and the early establishment of adaptive responses against SARS-CoV-2. Between 5 and 7 dpi, high levels of Th1/Th2 cytokines IL-2, IL-4, IL-10, and TNF-α were observed, reflecting local T cell responses. The late phase of infection (7 to 9 dpi) was characterized by high levels of inflammatory cytokines (IL-12, IL-15, GM-CSF, G-CSF, and TNF-α) and chemokines (MIP-1β, MCP-1, and IL-8), this last wave being associated with inflammation resolution [22,23,25,43,50].

Impaired or delayed cytokine signaling has been associated with very high risk of severe or fatal COVID-19. If the innate response delay is too long, as it has been observed in some individuals with defective type I IFN response [52,53], the priming and establishment of an efficient adaptive response is compromised, resulting in an ever-expanded innate response associated with severe disease [54]. In keeping with human studies, in NHPs, a delayed and more severe cytokine storm appears as a possible mechanism of severity in aged individuals [25,55]. In a study comparing the cytokines dynamics in young and aged RhM, it was observed that young macaques presented higher cytokine levels in the first week following SARS-CoV-2 infection, with a subsequent normalization to pre-infection levels. On the other hand, aged animals presented a delayed cytokine response, reaching higher levels at 2 weeks post-infection, which was associated with an unfavorable outcome [25]. In the two AGMs that developed ARDS, elevated levels of IL-1β, IL-4, IL-6, IL-8, IL-13, IFN-γ and TNF-α as well as delayed activation of adaptive immune responses seemed to correlate with disease severity [28].

**Figure 1:**
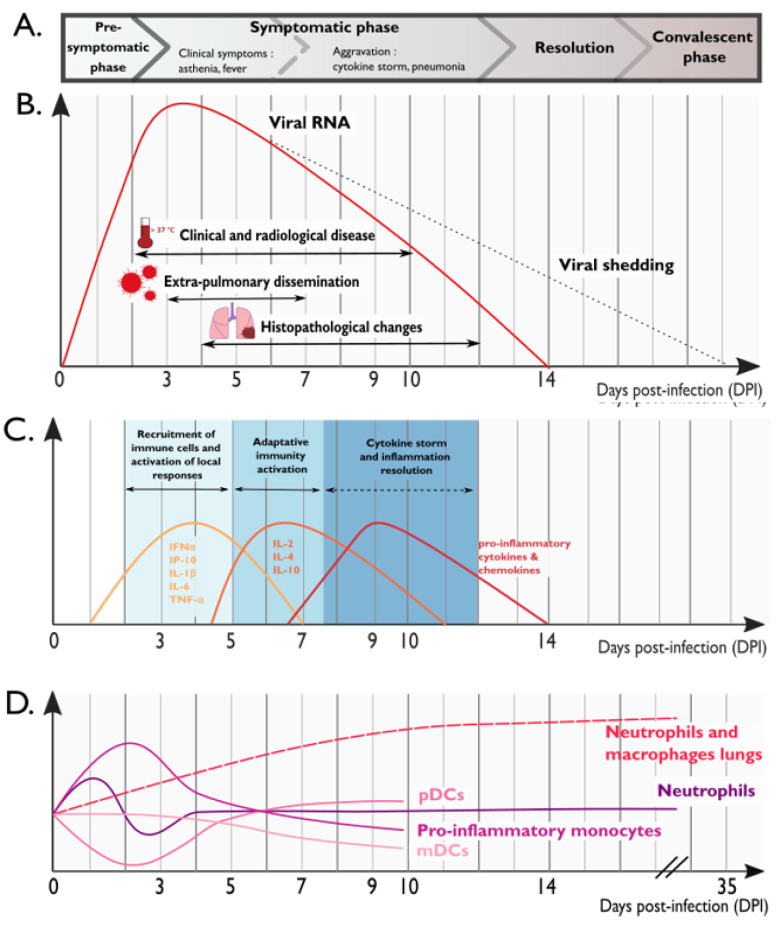

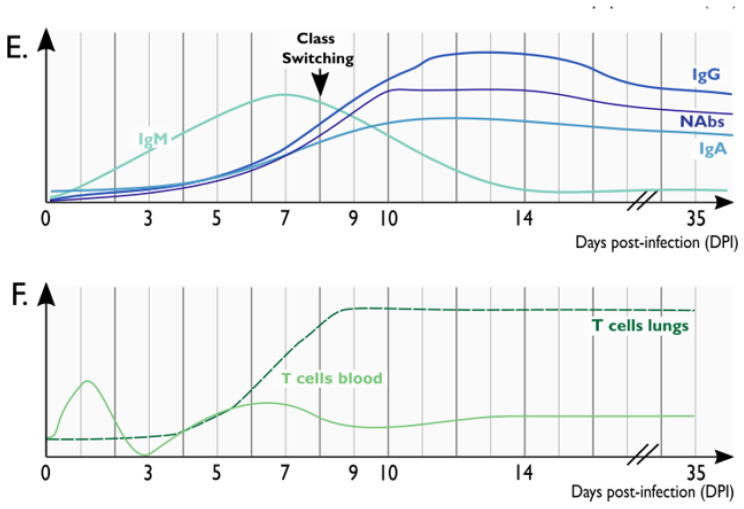
Schematic representation of the clinical, virologic and immunologic features observed over the course of SARS-CoV-2 infection in NHPs. (**A**) Clinical correlates of SARS-CoV-2 infection in NHPs. (**B**) SARS-CoV-2 viral kinetics and histopathological changes in the lungs of NHPs. (**C**) Cytokine production in the lungs of SARS-CoV-2 infected NHPs. (**D**) Dynamics of innate immune cells in the blood (solid lines) and in the lungs (dashed line). (**E**) Dynamics of virus-specific antibodies. (**F**) Dynamics of virus-specific T cells in the blood (solid line) and in the lungs (dashed line). Similar dynamics are observed in SARS-CoV-2 infected individuals. Figure was generated using Inkscape.

In conclusion, NHPs are suitable models for SARS-CoV-2 infection because they share more aspects of the human physiology than other animal models. Despite some limitations associated with disease severity, each of these species provides distinct insights for the study of SARS-CoV-2 pathogenesis, with implications for treatment and vaccine development. The global virologic and immunologic characteristics of SARS-CoV-2 pathogenesis in NHP models discussed in this section are summarized in Figure 1. The contribution of NHP models to the development of COVID-19 treatment and vaccine candidates are discussed in the following chapters.

## 3. Treatment for COVID-19: Contribution of NHP Models

Due to the impact of COVID-19 pandemic, speed of action to identify potent curative drugs and vaccines against SARS-CoV-2 is crucial. Normally, drug development requires several steps until its use in the clinical practice, which includes preclinical studies in animals. In the context of COVID-19, besides NHPs, other animal models, such as rodents, have been used to test potential drug candidates. Nevertheless, due to their close phylogenetic relationship with humans, NHPs appear to be the most suitable animal model to evaluate antivirals’ and monoclonal antibodies’ (mAb) pharmacokinetics (PK) and pharmacodynamics (PD). They can be used as a relevant tool for extrapolating drug doses and human PK, which may accelerate the research of drugs that will undergo clinical trials [56].

Several compounds against SARS-CoV-2 infection are currently being evaluated or were already validated in NHP models (Table 2 and Table 3). Combined with the principle of drug repurposing, efficacy evaluation in NHPs allows a rational prioritization of drug candidates [57]. Remdesivir (Veklury), the only antiviral against SARS-CoV-2 infection currently approved by the FDA [58], is the best example. This nucleotide analogue prodrug (GS-5734) was originally developed to treat Ebola virus infection and was previously shown to be effective against MERS-CoV and SARS-CoV infections [59,60,61]. Moreover, its PK was previously determined in RhM [59]. When administered early after infection, Remdesivir showed clinical benefits in reducing lung damage, despite not reducing viral shedding in the upper respiratory tract of SARS-CoV-2 infected RhM [40].

On the other hand, the antimalarial drug hydroxychloroquine (HCQ) drew controversies regarding its potential antiviral or clinical effect on SARS-CoV-2 infection. Whereas clinical studies reported contradictory results [57], an NHP study demonstrated the lack of efficacy of HCQ alone or combined with azithromycin against SARS-CoV-2 [50]. Notably, one advantage of NHP models is the possibility to control environmental, viral and host parameters, such as identical inoculum size, time pre- and post-infection, and treatment doses. Evaluation of HCQ in the NHP model was critical to determine the PK of the molecule and its compatibility with a potential antiviral activity into the lungs. It also showed that either prophylactic or therapeutic administration of HCQ at low or high dose, alone or combined with azithromycin, did not confer protection against SARS-CoV-2 infection in CyM [50]. This study evidenced a lack of HCQ efficacy in vivo and contributed to ruling out this drug as a treatment for COVID-19.

Blocking the interaction between SARS-CoV-2 envelope spike protein and its cellular receptor, notably ACE-2, is considered an effective strategy for the development of antiviral treatments. Macaques have been recognized among the most relevant animal models for the study of spike/ACE-2 interaction inhibitors [64]. Structural studies and docking simulations with SARS-CoV-2 spike protein showed that the interaction between the spike receptor-binding domain (RBD) and ACE-2 from CyM, ferret and Chinese hamster is comparable to the observed in humans [64]. On the contrary, mice, rats and guinea pigs seem unsuitable for such studies [64,65].

Another way to block spike/ACE-2 interaction is via monoclonal antibodies (mAbs), which have been tested in NHPs. For instance, REGN-CoV-2, a cocktail of two monoclonal antibodies (REGN10933/casirivimab and REGN10987/imdevimab) targeting non-overlapping epitopes on the SARS-CoV-2 spike protein has shown great antiviral potential on RhM [66]. Similarly, CB6 and LY-CoV555 (bamlanivimab), both mAbs derived from patients’ convalescent plasma, have shown great potency against SARS-CoV-2 infection in RhM models [67,68]. Of note, FDA issued an emergency use authorization (EUA) for LY-CoV555 and REGN-CoV-2 for the treatment of mild to moderate COVID-19 in adults and pediatric patients who are at high risk for progressing to severe COVID-19 [69]. The provisional analysis of LY-CoV555 phase 2 clinical trial (NCT04427501) showed mitigated results, while the initial analysis of REGN-CoV2 (NCT04425629) [70] demonstrated a superior effect in patients compared to LY-CoV555 [71]. However, administration of LY-CoV555 combined with another mAb, LY-CoV016 (etesevimab), promoted a statistically significant reduction in SARS-CoV-2 viral load [72]. Bamlanivimab in combination with etesevimab received FDA EUA [69].

Despite the encouraging results observed with mAbs in limiting viral entry, SARS-CoV-2 variants of concern (VOC) with mutations in the spike protein have recently emerged; therefore, the antiviral potential of these drugs against the emerging VOC is yet to be determined. To this regard, COVA1-18, a neutralizing antibody isolated from a convalescent patient, showed a strong antiviral activity in vitro, which was equally potent against the currently dominant D614G variant, as well as against the B.1.1.7 variant [73]. In vivo studies in CyM showed an important reduction in viral titers in the lungs, and a pre-exposure prophylaxis (PrEP) study confirmed the potent protective effect of COVA1-18 against SARS-CoV-2 infection [73]. The emergence of escape mutations in the spike following treatment with COVA1-18 was not observed. However, it was predicted to lose potency against variants harboring the E484K mutation [73]. Hence, these evidences highlight the need for mAbs cocktails targeting different epitopes to limit viral escape and the emergence of others VOC.

**Table 3:** Monoclonal antibody therapies against SARS-CoV-2 infection tested in NHP models.

mAb	Description	Animal Model Used in Preclinical Studies	Antiviral and Clinical Effects	Toxicity Data	Clinical Studies	Reference
REGN- COV2	Cocktail of two potent neutralizing antibodies (REGN10987+ REGN10933) targeting non-overlapping epitopes on the SARS-CoV-2 spike protein	RhM	Prophylactic administration led to strongly reduced viral load.	N/A	NCT04425629	[66]
LY-CoV555	Cocktail of two human IgG1 mAbs targeting different epitopes on the SARS-CoV-2 spike protein	RhM	Prophylactic administration led to lower viral loads and reduced viral shedding.	N/A	NCT04411628 NCT04427501 NCT04497987 NCT04501978	[68]
MW05/LALA	SARS-CoV-2 Spike glycoprotein RBD-targeting mAb	RhM	Potent therapeutic and prophylactic effect on SARS-CoV-2 infection and clinical disease.	None	N/A	[74]
COVA1-18	SARS-CoV-2 Spike glycoprotein RBD-targeting mAb	CyM, hACE2 mice, Syrian hamster	PreP in CyM led to strong protection, prophylactic administration led to potent reduction of viral load in the lungs.	N/A	None	[73]

N/A: not available; RhM: Rhesus Macaque; CyM: Cynomolgus Macaques.

In addition to mAbs, other class of molecules targeting the interaction between SARS-CoV-2 spike and ACE-2 is currently being evaluated. Among them, Dalbavancin, an approved lipoglycopeptide antibiotic, has yielded promising results in preclinical models [63]. Dalbavancin directly binds to human ACE-2 with high affinity, thereby blocking its interaction with the SARS-CoV-2 spike protein. In vivo functional antiviral studies in both RhM and humanized mice (hACE-2) confirmed the inhibition of SARS-CoV-2 replication in the lungs and evidenced a protection against pulmonary lesions. Reduced infiltration and lower levels of the cytokines/chemokines MCP-1 and IL-8 were observed in the lungs of infected animals [63]. As for mAbs, the efficacy of Dalbavancin against SARS-CoV-2 VOCs is yet to be determined.

Other innovative therapies are under evaluation to treat COVID-19, which is the case of immunomodulatory drugs. Because of the impact of inflammation and cytokine storm in the severity of COVID-19 pathogenesis, drugs aiming to treat the deregulation of inflammatory response are also under investigation (Table 2). Baricitinib (Olumiant), a clinically approved JAK1/2 inhibitor with potent anti-inflammatory properties, was recently shown to reduce immune activation and to limit cytokines and chemokines production by alveolar macrophages in RhM, evidencing a beneficial role for its application in severe disease [62]. The FDA approved Baricitinib, in combination with Remdesivir, for the treatment of COVID-19 in hospitalized adults and pediatric patients requiring supplemental oxygen, invasive mechanical ventilation, or extracorporeal membrane oxygenation. The treatment strategies tested in NHPs are listed in Table 2 and Table 3.

As discussed previously, most NHP models recapitulate mild but not severe disease. Although AGM and baboons were characterized to reflect a more severe pathogenesis [18,28], so far, RhM and CyM remain the major NHP models used in preclinical studies. Drug testing in AGM and baboons is yet to be investigated. Despite all efforts, validated treatment options for COVID-19 remain scarce.

In this context, some prophylactic and therapeutics interventions that showed promising results in preclinical studies, either in NHPs or other animal models, brought disappointing results in clinical trials [75]. In order to efficiently bridge the translational gap between fundamental and clinical studies, selecting a validated and predictive animal model is critical. In this regard, a better rationalization and harmonization of preclinical assays in terms of inoculum size, viral isolate, routes of administration, standardization of assays to evaluate antiviral effect and correlates of protection, for instance, could certainly accelerate research and limit the number of inconclusive studies [76].

## 4. Protective Immunity to SARS-CoV-2 Infection and Vaccination

The development of long-lasting immunity against SARS-CoV-2 infection, either by infection or vaccination, is the major hope to stop the COVID-19 pandemic and to limit the economic and public health consequences. On the one hand, some studies have described that immune memory against SARS-CoV-2 may last for several months after infection [77,78,79,80]. On the other hand, it is known that reinfections by common human coronaviruses occur, and a number of cases of SARS-CoV-2 reinfections were reported in patients that recovered from a previous infection [81,82,83,84,85,86]. Besides, following the emergence of VOC, increasing numbers of studies are reporting reinfections with these new variants [87,88,89]. This raises questions on the infectivity of different SARS-CoV-2 variants, as well as the duration of protective immunity, which is a crucial point in the perspective of global vaccination efficacy. 

In NHP models, it was reported that, following a primary exposure, RhM were protected against SARS-CoV-2 reinfection [44,48]. These animals had no detectable viral RNA in tissues, histopathological signs of interstitial pneumonia or pulmonary lesions. Robust humoral and cellular immune responses to natural infection were observed in these RhM. They developed anti-spike IgGs and neutralizing antibodies, which were enhanced by the second exposition to SARS-CoV-2. More recently, it was shown that relatively low antibody titers are sufficient for protection against SARS-CoV-2 in RhM, and that cellular immune responses may contribute to protection if antibody responses are suboptimal [47,90]. Altogether, these studies pointed to a key role of both humoral and cellular adaptive immunity generated upon primary exposition in the protection of these NHPs from reinfection. The characteristics of protective humoral and cellular immune responses elicited by natural infection and vaccination are discussed in the following sections.

### 4.1. Humoral Immunity to SARS-CoV-2 in NHP Models

The longitudinal kinetics and the magnitude of humoral immune responses against SARS-CoV-2 were assessed in different NHP species. Following SARS-CoV-2 infection, specific antibodies were elicited by 7–10 dpi. These virus-specific antibodies included IgM, IgG, and also IgA. In RhMs, a class switching from IgM to IgG was reported to occur between 7 and 14 dpi. IgM and IgG reached the highest levels at 14 and 28 dpi, respectively [46]. Later, in the convalescent phase, IgG, specially IgG1, was the predominant antibody class detected in the serum of RhM [44,45,48].

In addition to IgG and IgM, IgA antibodies appear to be key in mediating SARS-CoV-2–specific responses, particularly in the upper respiratory tract mucosa [91]. In RhMs, IgA is detected by 10 dpi [45]. Although IgA titers are usually lower than IgG titers in the serum, IgA was detectable in the convalescent phase in the serum of RhM [48].

Regarding antibody specificity, anti-spike responses seem to be predominant, but antibodies targeting other viral protein such as nucleocapsid were also identified in NHPs [44,45,47,48,92]. Nucleocapsid and spike IgG titers are often highly correlated. Spike is the target of SARS-CoV-2 neutralizing antibodies (NAbs), and mostly NAbs target the receptor-binding domain (RBD). In NHPs, the increase in antibody levels, especially NAbs, coincided with a decrease in viral load in nasopharynx and broncho-alveolar lavages [47,48]. 

Despite their protective role, high Ab titers are associated with higher antigen loads and severe disease. In NHPs, antibody levels were higher in older RhM and CyM, which could be linked to the age-related severity of infection in this species [18,90].

The protective efficacy of natural immunity against re-exposure to SARS-CoV-2 was reported in RhM [44,48]. Upon reinfection, NAb titers significantly increased, being associated with protection. To elucidate the relative importance of humoral immunity protection against SARS-CoV-2, IgG was purified from the plasma of convalescent RhM after reinfection. IgG was adoptively transferred to naïve animals and protected these recipient macaques against challenge with SARS-CoV-2 in a dose-dependent fashion, evidencing the crucial role of antibodies in mediating protection against viral infection and replication in the lungs [47].

Altogether, these studies confirm the importance of neutralizing antibodies in protecting NHPs against SARS-CoV-2 infection. Eliciting a sufficient humoral response seems crucial in the protection of individuals against SARS-CoV-2 for vaccination efforts.

### 4.2. T Cell Responses against SARS-CoV-2 in NHPs

It is well established that T cell responses have protective roles in controlling viral infections. In SARS-CoV-2 infected patients, CD4+ and CD8+ T cell responses have been mostly explored in the convalescent phase. In NHPs, the longitudinal dynamics of T cell responses have been well characterized.

SARS-CoV-2-specific CD4+ T cells can be detected as early as 3 dpi. In the blood, both CD4+IFN-γ+ Th1 and CD4+IL-4+ Th2 populations were observed early, but gradually decreased over the course of infection [27]. In the lungs, robust CD4+ and CD8+ T cell responses, characterized by the production of IFN-γ, IL-2 and Granzyme B, were detected early (3 dpi) and were maintained at later time points (9–21 dpi), further decreasing. There were no age-related differences in T cell responses in NHPs, although IL-2 expression on T cells was higher in young when compared with old RhM [22,27,45]. SARS-CoV-2 specific CD4+ and CD8+ T cells remained detectable at the convalescent phase of infection (35 dpi) in RhMs [48].

To evaluate the role of CD8+ T cells in contributing to protective efficacy against rechallenge, these cells were depleted in convalescent RhM prior to reinfection. Following SARS-CoV-2 rechallenge, virus was detectable in the lungs and nasal swabs of CD8-depleted animals. IFN-γ, TNF-α and IL-2 spike-specific CD8+ T cell responses were shown to contribute in protecting RhM against reinfection [47].

In another study, the authors explored the contribution of CD4+ and CD8+ T cells in pathogenesis and in protecting for reinfection in vivo [46]. Depletion of CD4+ T cells produced only a minimal impact on CD8+ T cell responses but had a significant negative impact on B cell responses. In the CD8-depleted group, CD4+ T responses to the second infection appeared slightly stronger than in controls, possibly as a compensatory response to the lack of CD8+ T cells. A delayed viral clearance was observed in the depleted animals in comparison to controls; however, RhM could control reinfection despite CD4+ and CD8+ T cell depletion prior to first encounter with SARS-CoV-2. Altogether, these results pointed to a major role of CD4+ and CD8+ T cells in the rapid resolution of acute SARS-CoV-2 infection, and evidenced the crucial role of CD4+ T cells in the development of humoral responses against SARS-CoV-2 [46].

Tfh cells are specialized providers of B cell help and are critical for the development of NAbs and long-term humoral immunity [54]. In NHPs, increased frequencies of CD4+Tfh were observed from 7dpi in the blood and specific CD4+ Tfh targeting nucleocapsid and spike antigens were detected in lymphoid tissues germinal centers (GC) [45]. In addition to helping antibody responses, these CD4+Tfh may also help the development of CD8+ responses, although it is still unclear in the context of SARS-CoV-2 infection. Other CD4+ populations also seem to play important roles in SARS-CoV-2 pathogenesis. Increases in CD4+Foxp3+ Treg cells were observed from 3 to 21 dpi, suggesting that these cells play a relevant role in controlling inflammation. Minor changes in the frequency of CD4+IL-17+ Th17 cells were observed in blood [22].

Altogether, these results give hope that the development of vaccines eliciting robust protective humoral and cellular immune responses might prevent infection and mitigate the morbidity and mortality caused by SARS-CoV-2 infection.

### 4.3. Vaccine Candidates against SARS-CoV-2 Infection: Preclinical Studies in NHPs

Nowadays, vaccination represents the main foreseeable strategy to contain COVID-19 pandemics. The global vaccine effort in response to this pandemic is unprecedented in terms of scale and speed. As of April 2021, 184 vaccine candidates against SARS-CoV-2 were undergoing preclinical studies, and 88 were in different phases of clinical trials. Among them, 27 were in clinical trials phases II/III, III or IV [93]. In this review, we focused on the vaccine candidates with available data of preclinical studies developed in NHPs that then reached human phase 3 clinical studies.

Conventional and innovative platforms were used in the development of COVID-19 vaccine candidates. The vaccines that are currently undergoing clinical trials phases III or IV are based on the following technologies: (i) inactivated virus (ex. Coronavac, BBIBP-CorV, and COVAXIN); (ii) nonreplicating adenovirus-based vectors (ex. ChAdOx1 nCov-19, Gam-COVID-Vac, Ad26.COV2.S); (iii) protein subunit (ex. NVX CoV2373, SCB-2019, ZF2001); (iv) RNA-based vaccines (mRNA-1273, BNT162b2 and CVnCoV Vaccine), and (v) DNA-based vaccines (INO-4800). Vaccines based on different technologies such as virus like particles (VLPs), replicating vectors or vectors associated with antigen presenting cells are currently in the early stages of clinical investigation.

The preclinical studies of these COVID-19 vaccine candidates were conducted in RhM, CyM and baboons (Table 4) with the objective to provide initial evaluation of vaccine performance and safety, and in some cases, to provide an indication about the dose to be used in clinical trials. The immune responses elicited by vaccination, i.e., antibody titers, neutralizing activities and T cell responses, were assessed in different studies, as well as the clinical features of SARS-CoV-2 infection and the systemic and tissue viral loads after a challenge with a SARS-CoV-2 isolate. Vaccine efficacy was evaluated based on protection from SARS-CoV-2 infection and in the capacity to limit viral shedding. 

Overall, the vaccine candidates listed in Table 4 led to a strong production of anti-SARS-CoV-2 antibodies following vaccination, and pointed to neutralizing antibodies as the major correlate of protection [94,95,96,97,98,99,100,101,102,103,104,105,106,107]. Whereas humoral responses were broadly induced by all vaccine candidates tested in NHPs, the induction of cellular immune responses was heterogeneous, which seems to be dependent on the vaccine platform. CD4+ T cell responses were induced by most vaccine candidates, whereas CD8+ T cell responses were infrequently observed in these preclinical studies in NHPs, when assessed [95,98,99,100,102,103,104,105,106,107]. In general, T cell responses were Th1 polarized characterized by IFNγ production. The cytokine profile of Th2-biased responses, which might be linked with vaccine-associated enhanced respiratory disease (VAERD), was rarely seen.

**Table 4:** NHP preclinical evaluation of SARS-CoV-2 vaccines that have reached phase 3 of clinical studies.

Vaccine Manufacturer	Vaccine Platform	NHP Species Used in Preclinical Studies	Phase 3 Clinical Studies	Immune Responses Elicited by Vaccination in Preclinical and Clinical Studies *	Reference
PiCoVacc/CoronaVac Sinovac	Inactivated	RhM	NCT04456595 669/UN6.KEP/EC/2020 NCT04582344 NCT04617483	IgG, NAb	[94,108]
BBV152/COVAXIN Bharat Biotech	Inactivated	RhM	NCT04641481 CTRI/2020/11/028976	IgG, NAb	[96,109,110]
BBIBP-CorV Beijing Institute of Biological Products/Sinopharm	Inactivated	RhM/ CyM	ChiCTR2000034780 NCT04560881	NAb	[101,111]
Inactivated SARS-CoV-2 Vaccine Institute of Medical Biology + Chinese Academy of Medical Sciences	Inactivated	RhM	NCT04659239	IgG, NAb, T cells (IFNγ)	[102,112]
ChAdOx1 nCov-19 University of Oxford/AstraZeneca	Non-replicating viral vector (ChAdOx1-S)	RhM	ISRCTN89951424 NCT04516746 NCT04540393 CTRI/2020/08/027170	IgG, NAb, T cells (IFNγ)	[99,113]
Ad26.COV2.S Janssen Pharmaceutical	Non-replicating viral vector (Ad26)	RhM	NCT04505722 NCT04614948	NAb, Th1	[106,114]
mRNA-1273 Moderna/NIAID	RNA-based	RhM	NCT04470427	IgG, NAb, TCD4 (Th1), Tfh	[103,115]
BNT162b2 BioNTech/Fosun Pharma/Pfizer	RNA-based	RhM	NCT04368728	IgG, NAb, TCD4 (IFNγ, IL-2, TNFα), TCD8 (IFNγ)	[100,116]
CVnCoV CureVac AG	RNA-based	RhM	NCT04674189	IgG, NAb, T cells (IFNγ)	[107,117]
INO-4800 InovioPharmaceuticals/International Vaccine Institute	DNA-based	RhM	NCT04642638	IgG, NAb, T cells (IFNγ, TNFα)	[95,97]
NVX CoV2373 Novavax	Protein subunit	CyM/ Baboon	2020-004123-16 NCT04611802	IgG, NAb, TCD4 (IFNγ, IL-2, TNFα)	[98,104,118]
SCB-2019 Clover Biopharmaceuticals/GSK/ Dynavax	Protein subunit	RhM	NCT04672395	IgG, NAb	[105,119]
ZF2001 Anhui Zhifei Longcom Biopharmaceutical + Institute of Microbiology, Chinese Academy of Sciences	Protein subunit	CyM/RhM	NCT04646590	IgG, NAb, T cells (IFN-γ, IL-2, IL-4)	[120,121]

Ad: Adenovirus; RhM: Rhesus Macaque; CyM: Cynomolgus Macaques; IgG: Immunoglobulin G, NAb: Neutralizing Antibodies, TCD4: CD4+ T-lymphocytes, TCD8: CD8+ T-lymphocytes, Th1: CD4+ T-lymphocytes helper type 1. * The immune responses elicited by vaccination listed in Table 4 are those described in the original reports, although we cannot rule out that other immune responses were induced, but were not assessed in these studies.

All these vaccine candidates succeeded in inducing protective immune responses against SARS-CoV-2 in NHPs despite the differences regarding technology, dose (concentration and number of doses required to elicit robust immune responses), prime-boost strategy and route of administration [93]. Comparing the efficacy of these vaccines is beyond the scope of this review, but it is important to take into account that the study design of these preclinical studies varied in terms of (i) challenge virus stock (SARS-CoV-2 isolate, dose and route of inoculation), (ii) time between vaccination and challenge, (iii) the immunoassays used to quantify total and neutralizing antibodies, and to characterize the T cell responses induced upon vaccination (See [122] for a critical review). The study design and the main findings of the preclinical studies conducted in NHPs listed in Table 4 are briefly detailed in the following sessions.

#### 4.3.1. Inactivated Virus Vaccines

Inactivated virus vaccines are produced by growing SARS-CoV-2 in cell culture, followed by chemical inactivation of the virus, and are often adjuvanted [123]. These were among the first COVID-19 vaccines to undergo preclinical and clinical studies. The immunogenicity of the Sinovac PiCoVacc/CoronaVac vaccine was first evaluated in RhM. Two vaccine doses (3 or 6 μg) were tested in groups of four RhM that were immunized on days 0, 7, and 14, and were challenged intratracheally with SARS-CoV-2 (strain CN1) on day 22. Spike-specific IgG and NAb increased from week two of post-vaccination, reaching higher levels at week three. Although vaccination did not prevent infection, it protected from severe lung disease, and virus clearance from pharynx or lungs at 7 dpi was observed among the high vaccine dose group [94].

The BBV152/COVAXIN vaccine was evaluated in RhM. Three vaccines formulations were tested: BBV152A (3 μg+alum+imidazoquinoline), BBV152B (6 μg+alum+imidazoquinoline), and BBV152C (6 μg+alum). RhM were vaccinated on days 0 and 14 intramuscularly and were challenged with SARS-CoV-2 (intratracheally and intranasal with the NIV-2020-770 isolate) fourteen days after receiving the second dose. Increasing SARS-CoV-2 specific IgG and NAb titers were observed from week three post-vaccination. Viral RNA was detected in vaccinated animals early after infection, but viral clearance was observed from 7 dpi. No evidence of pneumonia or histopathological abnormalities was observed in the vaccinated groups. The formulation BBV152A (3 μg+alum+imidazoquinoline) showed higher NAb titers post-vaccination and was chosen for clinical studies [96].

The immunogenicity and toxicity of the BBIBP-CorV vaccine (Sinopharm/Beijing Institute of Biological Products) were evaluated in RhM and CyM, respectively. Two vaccine doses (2 or 8 mg) were tested in groups of four RhM that were immunized intramuscularly on days 0 and 14, and were challenged intratracheally with SARS-CoV-2 (SARS-CoV-2/WH-09/human/2020/CHN isolate) 10 days after the second immunization. NAb increased following vaccination. Vaccination led to lower (low-dose group) or undetectable (high-dose group) viral loads in the throat and anal swabs during the first days following challenge, and at 7 dpi viral load was undetectable in the lungs of all vaccinated RhM. Lung pathology was also prevented or reduced in the vaccine groups. No abnormalities or adverse effects were observed in the long-term toxicity analyses conducted in CyM [101].

The immunogenicity of the inactivated SARS-CoV-2 vaccine developed by the Institute of Medical Biology + Chinese Academy of Medical Sciences was evaluated in RhM. Groups of 3–4 RhM were inoculated intramuscularly with three vaccine doses (20, 100 or 200 ELISA units, EU) on days 0 and 14. Animals were then challenged via nasal route with SARS-CoV-2 (KMS-1 isolate; GenBank No: MT226610.1). Increased Nab titers were observed in vaccinated animals 7 days after receiving the booster injection in a dose-dependent manner. In addition, this vaccine induced IFNγ production by T cells and antibodies against diverse viral proteins. After challenge, lower viral load levels were observed in nasal, pharyngeal and anal swabs of vaccinated RhM when compared with the placebo group and also in tissues at the time of euthanasia. Similar to the observed for the other inactivated virus-based vaccines, a protective effect of the lung histopathology was reported [102].

#### 4.3.2. Non-Replicating Viral Vector Vaccines

The adenovirus-vector-based vaccine ChAdOx1 nCoV-19, which encodes a nonstabilized form of the SARS-CoV-2 spike protein, was first evaluated in RhM. Groups of six RhM were vaccinated intramuscularly once (day 0) or in a prime-boost protocol (days 0 and 28) with 2.5 × 10^10^ ChAdOx1 nCoV-19 virus particles each. Animals were then challenged with SARS-CoV-2 (WA1-2020 isolate; GenBank No: MN985325.1) intratracheally, intranasally, orally and ocularly 28 days after receiving the single-dose or the boosted-injection. Anti-spike IgG and NAb increased after vaccination, and the second dose boosted these responses. All ChAdOx1-vaccinated macaques became infected following challenge, but they presented a better clinical score, less lung damage, and lower viral loads when compared with the control group. Based on these data, the prime-boost strategy was chosen for clinical trials [99].

The Janssen Ad26.COV2.S vaccine candidate was also evaluated in RhM. The authors first explored the immunogenicity of seven Ad26 vector constructions expressing modified SARS-CoV-2 spike protein. Groups of 4–6 RhM were immunized in a single-shot vaccine strategy by the intramuscular route and were challenged with SARS-CoV-2 (USA-WA1/2020 isolate) by the intranasal and intratracheal routes six weeks post-immunization. NAb were detected in vaccinated animals from week 2 post-vaccination and increased at week 4. Cellular immune responses were characterized by IFNγ secretion with minimal or no IL-4 responses, suggesting Th1-biased responses. The optimal Ad26 vaccine induced robust NAb responses and provided complete or near-complete protection in bronchoalveolar lavage (BAL) and nasal swabs after SARS-CoV-2 challenge. These data pointed to vaccine-elicited NAb as the major correlate of protection. The optimal Ad26 vector-based vaccine for SARS-CoV-2 (Ad26.COV2.S) was then evaluated in clinical trials [106]. Despite the advantages of a single-dose vaccine strategy, a two-dose Ad26.COV2.S regimen induced higher peak binding and neutralizing antibody responses compared to a single dose in NHPs [124]. These results supported the development of a phase 3 clinical trial to evaluate the two-dose strategy and to compare with the one-dose trial.

#### 4.3.3. RNA-Based Vaccines

The COVID-19 pandemic paved the way for the large-scale use of RNA-based vaccines. The Moderna mRNA-1273 vaccine, which encodes the pre-fusion stabilized spike protein of SARS-CoV-2, was evaluated in RhM. Animals were vaccinated intramuscularly at week 0 and at week 4 with either 10 or 100 μg of mRNA-1273 and at week 8 they were challenged with SARS-CoV-2 (USAWA1/2020 strain) by the intratracheal and intranasal routes. Specific anti-spike IgG and NAb activities increased in a dose-dependent manner following vaccination, in particular after the second dose. Vaccination induced a dose-dependent Th1–biased CD4+ T cell responses and IL-21 producing Tfh, but low or undetectable Th2 or CD8+ T cell responses were observed among RhM vaccinated with mRNA-1273. Following challenge, viral replication was not detectable in BAL by day 2 in both 10 or 100 μg vaccinated groups. In the upper respiratory tract, no viral replication was detectable in the nose of the RhM receiving the 100 μg dose by day 2 after challenge. Little or no signs of lung pathology were observed in the high dose group. Importantly, the vaccine scheme and dose assessed in the Moderna preclinical trials in RhM were directly translated to the clinical trials in humans [103], underlining the critical relevance of the preclinical studies using NHPs in the context of SARS-CoV-2 vaccine development.

The immunogenicity of the Pfizer/BioNTech BNT162b2 vaccine, which encodes the full-length transmembrane spike glycoprotein locked in its prefusion conformation, was evaluated in groups of six RhM. Animals were immunized intramuscularly on days 0 and 21 with 30 μg or 100 μg of BNT162b2. Increased levels of IgG and NAb were observed at day 14 post-vaccination and augmented following the second dose. CD4+ T cells producing IFNγ, IL-2 or TNF and CD8+ T cells producing IFNγ were induced upon vaccination. A low frequency of IL-4 producing-CD4+ T cells was observed. Forty-one to 55 days after the second dose, 6 RhM that were immunized with 100 μg of BNT162b2 were challenged with 1.05 × 10^6^ plaque-forming units of SARS-CoV-2 (strain USA-WA1/2020) through intranasal and intratracheal routes. Viral RNA was not detected in the BAL, in the nasal, oropharyngeal or anal swabs of vaccinated animals. No signs of lung disease were observed in these RhM, whether immunized or not [100]. Immunization of RhM with BNT162b2 provided evidence for protection of the lower respiratory tract, supporting a large-scale use in clinical trials [116,125].

The CVnCoV/CureVac vaccine is based on non-chemically modified mRNA encoding for full-length pre-fusion stabilized spike protein. Groups of six RhM were immunized intramuscularly with 0.5 μg or 8 μg of CVnCoV on days 0 and 28. Significant increase in IgG, NAb titers and spike-specific IFNγ producing cells were observed, especially after the second vaccination with 8 μg of CVnCoV, but not with 0.5 μg (suboptimal dose). Following the challenge with SARS-CoV-2 (Victoria/1/2020 isolate) through tracheal and nasal routes, reduced levels of total viral RNA in the upper and lower respiratory tracts were observed among the group that was immunized with 8 μg of CVnCoV. A significant reduction in the severity of lung lesions was also observed in the 8 μg CVnCoV vaccinated animals. These results showed that CVnCoV is safe and immunogenic in RhM, eliciting both humoral and cellular immune responses [107]. These findings were in agreement with the results of phase I clinical trials [117]. Altogether, these studies gave support for the evaluation of CVnCoV in a phase 2b/3 clinical trial [117].

#### 4.3.4. DNA-Based Vaccines

The INOVIO’s INO-4800 is based on a full-length SARS-CoV-2 spike DNA sequence optimized to enhance expression and immunogenicity. Preclinical trials in NHPs were conducted in a group of five RhM, immunized with INO-4800 (1 mg) at weeks 0 and 4. For this vaccine, immunization was through intradermal route, accompanied by electroporation. IgG titers against the full-length and different regions of the SARS-CoV-2 spike protein were detected following vaccination. NAb levels were also increased. Besides, T cell responses, as measured by IFNγ upon stimulation with SARS-CoV-2 peptide pools, were also increased following vaccination with INO-4800. Animals were challenged 3 months post-vaccination with SARS-CoV-2 (isolate USA-WA1/2020) by intranasal and intratracheal routes for the evaluation of long-term memory responses induced by vaccination. Both humoral and cellular responses expanded following challenge, which conferred protection as measured by lower viral load levels in the lungs and nasal swabs [95]. The INO-4800 DNA-based vaccine and the intradermal + electroporation immunization system showed safe in NHPs and were validated for evaluation in clinical trials [97]. 

#### 4.3.5. Protein Subunit Recombinant Vaccines

The Novavax NVX-CoV3273 is a subunit vaccine constructed from the full-length spike-protein and produced in the established baculovirus *Spodoptera frugiperda* (Sf9) insect cell expression system. The first immunogenicity study evaluated 1 μg, 5 μg, and 25 μg of NVX-CoV2373 with 50 μg of Matrix-M adjuvant administered intramuscularly on days 0 and 21 in baboons. Anti-spike IgG and NAb titers were detected following the first immunization, and importantly increased after booster injection. Receptor-blocking antibody titers were low after first injection, but significantly increased after the second immunization. High frequency of IFN-γ secreting cells (measured by ELISpot assay) and IFN-γ+, IL-2+, and TNF-α+ CD4+ T cells (measured by flow cytometry) were observed in those animals immunized with 5 μg or 25 μg of NVXCoV2373. IL-4 secretion was low in vaccinated animals [98].

This vaccine was then evaluated in CyM. Based on their prior experience in baboons, antigen (5 μg and 25 μg) and adjuvant (50 μg) dose levels were selected. Groups of 4 CyM were immunized with NVXCoV2373 intramuscularly on days 0 and 21. The immune responses elicited by vaccination in CyM had the same pattern as the ones observed in baboons. CyM were challenged with SARS-CoV-2 (2019-nCoV/USA-WA1/2020 isolate) via intranasal and intratracheal routes two weeks post-boost. Immunized animals had no detectable viral RNA in BAL and viral swabs two and four days post-challenge. Little or no signs of lung inflammation were observed in vaccinated animals [104]. NVX-CoV2373 vaccine appears to protect the upper and lower respiratory tracts, thus supporting clinical investigation.

Another protein subunit vaccine evaluated in NHPs was the SCB-2019. It consists in a platform technology named Trimer-Tag, which has an affinity purification scheme that allows a rapid production of a native-like pre-fusion form of trimeric SARS-CoV-2 spike (S)-protein subunit antigen in mammalian cells. Groups of six RhM were vaccinated intramuscularly on days 0 and 21 with 30 μg S-Trimer adjuvanted with 0.25 mL AS03, or 30 μg S-Trimer adjuvanted with 1.5 mg CpG 1018 plus 0.75 mg alum. High levels of binding and NAb titers were observed in both groups receiving adjuvanted S-Trimer. Titers increased after boost. Increases in the NAb were more prominent in the AS03-adjuvanted S-Trimer group. The vaccine efficacy was evaluated following challenge with SARS-CoV-2 virus (strain 107, China) intratracheally and intranasally on day 35. Vaccinated macaques presented a better clinical score, with no weight loss, no increase in body temperature and normal biochemistry parameters when compared with control group. Viral load was undetectable in the lungs 5 and 7 dpi. A trend for lower viral loads was observed in throat swabs, anal swabs, and tracheal brushes 1, 3 5 and 7 dpi. Lung histopathological analyses confirmed the reduced SARS-CoV-2 infection in animals vaccinated with S-Trimer [105]. The results of preclinical studies and the phase I clinical studies showed that both AS03 or CpG/alum adjuvanted vaccine formulations were immunogenic and well tolerated, thus were suitable for further clinical development.

The ZF2001 protein subunit vaccine candidate contains a dimeric form of the receptor-binding domain of the SARS-CoV-2 spike protein as the antigen, with alum-based adjuvant. Immunogenicity was evaluated in groups of 10 CyM that were immunized intramuscularly with 25 μg or 50 μg of ZF2001 vaccine on weeks 0, 4, 8 and 10. Immunization elicited RBD-binding IgG and NAb and titers increased following the second boost injection. The third and fourth boosts did not significantly increase IgG and NAb titers. The cellular immune responses were evaluated based on the IFN-γ, IL-2 and IL-4 production by stimulated cells. An enhanced Th1/Th2 balanced cytokine production was reported. To assess the protection efficacy, groups of 3 RhM were vaccinated with either 25 μg or 50 μg on days 0 and 21. Animals were then challenged at day 28 post-vaccination with SARS-CoV-2 (20SF107 strain) via intratracheal route. Both doses of ZF2001 protected from infection in lung, trachea and bronchus, and prevented lung lesions [121]. The use of the 25 μg dose in a three-dose schedule was chosen to be evaluated in a phase 3 trial for large-scale evaluation of ZF2001’s safety and efficacy [120].

### 4.4. Importance of Mucosal Immune Response Induced by Vaccination

Despite the diversity of vaccine platforms developed against COVID-19 infection and the differences in the study design of these preclinical studies conducted in NHPs, a common point was the fact that these vaccines often induced a protection of the lower airways. Nevertheless, most vaccines failed to induce sterilizing immunity in the upper respiratory tract, which suggests that although protecting from symptomatic disease, these vaccines might still enable SARS-CoV-2 transmission [123]. This raised the question about the importance of inducing IgA production in the upper respiratory tract to limit viral replication and transmission.

It was observed that live attenuated and replicating viral vectors were more likely to induce IgA in the upper airways than other vaccine platforms [123]. However, the RNA-based vaccine mRNA-1273 induced both IgG and IgA in the BAL of vaccinated RhM, which was associated with limited viral replication in BAL fluid and with the absence of subgenomic viral RNA in the upper airways [103]. The induction of anti-SARS-CoV-2 IgA in the in the upper airways by the different vaccine candidates and the role of these antibodies in protection from infection and onward transmission of SARS-CoV-2 was poorly explored and needs additional investigation.

Besides the vaccine platform, the inoculation route may also determine antibody production following vaccination [123]. Whereas intramuscular or intradermal vaccination leads to a predominant induction of serum IgG, intranasal or oral vaccination can efficiently induce mucosal antibody responses. We are tempted to consider that the combination of both approaches might favor sterilizing immunity in the upper respiratory tract. With this goal, several intranasal vaccine formulations that could stimulate IgA production are currently under investigation. 

Two vaccine candidates administered through different routes of inoculation were already tested in NHPs and showed suitable protection against a SARS-CoV-2 challenge [126,127]. One study combined subcutaneous prime followed by oral boosts of an adenovirus-5 vaccine platform. The hAd5 121S-Fusion + N-ETSD vaccine was designed to induce both humoral and enhanced Th1 dominant T-cell responses. They observed that two oral boosts induced strong responses that protected the upper and lower respiratory tracts from high titers of SARS-CoV-2. Importantly, in the context of SARS-CoV-2 vaccination, an oral boost presents a greater potential for generating mucosal immunity, particularly in the gastrointestinal tract, which is an important site for viral replication [126]. 

The other study evaluated the potential of intranasal vaccination with the ChAdOx1 nCoV-19—because intramuscular administration of this vaccine protected RhM from pneumonia—but did not reduce viral shedding. Here, the authors showed that intranasal vaccination of RhM resulted in robust immune responses; in particular, IgA and IgG were detected in nasal mucosal fluid and in BAL. This was associated with reduced shedding and a reduction in viral load in BAL and lower respiratory tract tissue [127]. 

Overall, these studies suggest that oral/intranasal vaccination (prime+boost or only boost) can induce immune responses comparable to subcutaneous/intramuscular administration, with a greater potential to limit SARS-CoV-2 transmission. Considering the impact of the SARS-CoV-2 pandemic, oral and intranasal vaccinations appear as alternatives to hypodermic injection to deliver vaccine and help controlling viral spread, especially at the large scale.

### 4.5. Emergence of SARS-CoV-2 Variants of Concern and Vaccination 

The emergence of variants with the capacity to escape from current vaccines and therapies targeting the spike protein raises questions on the potential of these vaccines to contain viral spread and to end up the COVID-19 pandemic. To date, most preclinical studies in NHPs were published before the emergence of these widely spread VOC; therefore, the contribution of NHPs in understanding the impact of VOC in SARS-CoV-2 pathogenesis and vaccine efficacy is limited. 

The mutation D614G was the first to be described in February 2020, and today most SARS-CoV-2 variants circulating worldwide harbor this signature. This mutation enhances the cleavage of the spike protein, a necessary step to the viral infection, which increases infectivity [128]. Several vaccine candidates showed capable to neutralize viruses harboring D614G mutation. In NHPs, Patel et al. demonstrated the ability of the INO-4800 DNA-based vaccine to protect against the D614G variant [95]. Other studies in NHPs also observed potent neutralization of vaccinated macaques against a variant with the D614G mutation [105,124,129]. Brouwer et al. investigated the emergence of SARS-CoV-2 mutants in NHPs, and they did not identify any mutant virus capable to escape antibody neutralization [130].

In April 2021, four VOC have been closely monitored due to potential impact on vaccine efficacy: the variants B.1.1.7 (Alpha, United-Kingdom), B.1.351 (Beta, South Africa), P.1 (Gamma, Brazil) and B.1.617.2 (Delta, India). The impact of some of these VOC on disease pathogenesis was evaluated in NHPs. The impact of B.1.1.7 (alpha) variant was investigated in AGM [131]. Significantly higher levels of viral RNA and infectious virus were found in the respiratory tract samples and tissues from B.1.1.7 infected animals, whereas D614G infected AGM showed significantly higher levels of viral RNA and infectious virus in rectal swabs and gastrointestinal tract tissues. Overall, B.1.1.7 infection in AGM exhibits increased respiratory replication and shedding, but without disease enhancement [131]. Another study investigated the pathogenicity of B.1.1.7 and B.1.351 variants in RhM [15]. The B.1.1.7 VOC behaved similarly to the D614G with respect to clinical disease, virus shedding and virus replication in the respiratory tract. However, the B.1.351 isolate resulted in lower clinical scores as a result of lower virus titers in the lungs, less severe histologic lung lesions and less viral antigen detected in the lungs. These subtle differences in the pathogenicity of B.1.1.7 and B.1.351 variants suggest SARS-CoV-2 evolution favors transmissibility and immune evasion rather than an increase in intrinsic pathogenicity [132].

The efficacy of the mRNA-1273 vaccine against the variants B.1.1.7 and B.1.351 was evaluated [133,134]. Serum from vaccinated NHPs was assayed for neutralization in vitro against pseudo-viruses containing mutations in the spike. Neutralization of the variant B.1.1.7 was similar to wild-type SARS-CoV-2. However, neutralization titers against the variant B.1.351 were lower than the observed for wild-type and B.1.1.7. In spite of this, the levels appear to be sufficient to protect individuals against infection [133]. In vivo evaluation of mRNA-1273 against SARS-CoV-2 B.1.351 infection was performed in RhM. The results showed that immunization with two doses of mRNA-1273 achieves effective immunity that rapidly controls lower and upper airway viral replication against the B.1.351 variant [134]. Results from clinical trials point to an impact of the B.1.351 and the P.1 variants in the ChAdOx1 nCov-19/AZD1222 efficacy [135]. 

To date, no data from ChAdOx1 nCov-19 vaccinated NHPs and SARS-CoV-2 variants are available. Further studies are needed to investigate the impact of emerging SARS-CoV-2 variants in the vaccine candidates tested in NHPs.

Altogether, these results imply the need for a continuous genomic surveillance to monitor viral evolution and the emergence of new SARS-CoV-2 variants with potential to aggravate disease escape the immune responses elicited by vaccination. This reveals the urgency for a global vaccination strategy to contain the COVID-19 pandemic. 

## 5. Conclusions

In this review we discussed the relevance of NHP models of SARS-CoV-2 infection and their contribution to the development of effective treatment and vaccine candidates to battle the COVID-19 pandemic. Studies in NHPs offer several advantages, often associated with the similarity of human and NHP immune systems and the possibility to perform the studies under standardized conditions. However, we must keep in mind that studies in NHPs present some limitations; therefore, NHPs are not always the best animal model for every aspect of the disease. In addition, as COVID-19 vaccine, therapies, and drug development have moved forward at an unprecedented pace, there is a current shortage of NHPs worldwide, especially RhM. This might outstrip the supply for COVID-19 research and for other biomedical research studies, pushing the scientific community to look for alternatives as the use of other species (baboons, AGMs) or other animal models.

In conclusion, the characterization of SARS-CoV-2 pathogenesis and immune responses in NHPs has proven comparable with the characteristics of the infection in infected patients, which favors the immediate translation of the results obtained to guide treatment and vaccine candidate tests in humans, which is key to fight the COVID-19 pandemics.

## Figures and Tables

**Table 1:** Characteristics of SARS-CoV-2 infection in different non-human primate species.

NHP Species	General Status	Viral Replication and Shedding	Histopathological Changes	Impact of Age on Disease	Immune Responses after Challenge	Reference
Common Marmoset	Inconstant and slight fever	Transient and low levels of viral RNA in swab samples	None	N/A	No virus-specific antibodies	[23]
Cynomolgus Macaques	Fever and body weight loss, chest radiography abnormalities	High and persistent levels of viral RNA in respiratory tract, fecal shedding and viral presence in digestive tract and spleen	Diffuse alveolar damage	Higher and more persistent viral titers	Virus-specific antibodies with neutralizing activity, T cell responses	[21,25,29]
Rhesus Macaques	Altered general status, fever, body weight loss and severe chest radiography abnormalities	Viral titer in respiratory tract, fecal shedding, viral presence in digestive and urinary tracts	Diffuse alveolar damage, mild changes in spleen and lymph nodes	More severe chest radiography abnormalities, higher viral titers in respiratory tract and severe interstitial pneumonia. Transcription dysregulation of inflammatory pathways and delayed cytokine storm	Virus-specific antibodies with neutralizing activity, T cell responses	[23,25,26,28,29]
African Green monkeys	Transient fever and loss of appetite, mild decrease of partial O_2_ pressure, possibility of digestive disease	Viral titers in respiratory tract and prolonged fecal shedding	Diffuse alveolar damage to severe interstitial pneumonia	Increased inflammatory cytokines, pathological lesions in lungs characteristic of ARDS	Virus-specific antibodies with neutralizing activity, T cell responses	[19,28,29]
Baboons	Body weight loss	Long-term viral persistence in respiratory tract and prolonged fecal shedding	Diffuse alveolar damage and interstitial pneumonia, rhinitis and tracheitis	Higher and more persistent viral titers	N/A	[23]

N/A: not available; ARDS: acute respiratory distress syndrome.

**Table 2:** Repurposed drugs against SARS-CoV-2 infection tested in NHP models.

Drug	Category/ Mechanism of Action	Animal Model Used in Preclinical Studies	Antiviral and Clinical Effects	Toxicity Data	Clinical Studies	Reference
Remdesivir (GS-5734)	Nucleotide analogue/ Viral RNA replicase Inhibitor	RhM	Lower virus titers in the lung, but no effect on viral shedding. Reduction of clinical signs of disease and lung tissue damage.	None	NCT0428070 NCT04292730	[61]
HCQ	Immunomodulator/ Undetermined (may inhibit viral transport in endosomes)	CyM	Lack of in vivo antiviral activity. No clinical efficacy, regardless the timing of treatment initiation and dose.	None	NCT04381936 NCT04315948	[50]
Baricitinib	Immunomodulator/ Selective JAK1/2 Inhibitor	RhM	No antiviral effect. Reduction of inflammation, decreased infiltration of inflammatory cells in the lungs, reduced NETosis activity, and more limited lung pathology.	None	NCT04401579NCT04421027	[62]
Dalbavancin	Lipoglycopeptide Antibiotic	RhM	Reduction of lung tissue damage. Lower virus titers and viral loads in the lungs. Reduction of IL-8 and MCP-1 in lung tissues.	None	N/A	[63]

N/A: not available; HCQ: Hydroxychloroquine; RhM: Rhesus Macaque; CyM: Cynomolgus Macaques.

## Data Availability

Not applicable.
